# The Courts and County Medical Societies

**Published:** 1863-10

**Authors:** 


### CORRESPONDENCE.

### THE COURTS AND COUNTY MEDICAL SOCIETIES.

To the Editor Buffalo Medical and Surgical Journal :

Sir:—In the last (September) number of your valuable Journal you have an editorial advocating the abolition of the Erie County Medical Society, in the closing sentence of which you challenge the defenders of the Society to "send in their copy." Though among the youngest of her knights, and perhaps the weakest, I pick up the gauntlet, and will at least show my devotion by shivering a lance in her favor.

You commence, in a very impersonal vein, to discuss the legal status of the County Societies of this State, but soon dropping this mood you make the Erie County Society do duty for the whole, and, as you advocate its honorable sepulture, I suppose you mean to write *requiescat in pace* over all of them. Let me restore the discussion to its original broad basis, and proceed to examine the question from this stand-point. I do this the more readily, because you claim to be contending for a principle, and not for a peisonal pique.

Now in the outset you say in effect that these Societies were formerly useful and honorable institutions, rightly endowed with the power to regulate their memberships and prescribe the rules of honorable professional standing. There, Sir, I am happy to agree with you, and beg leave to start the discussion from this common ground. If we are to advocate the abandonment by the profession of these Societies, which for half a century have been considered its honor and safeguard, it behooves us for the honor and dignity, no less than for the learning and usefulness of our calling, to make sure that we do so upon good and sufficient grounds. After a careful reading of your article I can find but two reasons alleged for this proposed dissolution, which I take the liberty to condense, as follows:

1.—"The Courts have opened the door of admission so wide, and protected it so feebly that the Society is made only the medium whereby quackery may obtain better company."

2.—Therefore these Societies are become useless, and "protect no interest, redress no wrong, cultivate no medical or other science, make now no dividing line between legitimate, honest and honorable medical practice and the grossest forms of pretension and quackery."

Now this, (which I believe a fair digest of you argument,) is a clear *non sequitur*, and reminds me of,

*Major*—Omnes Parisii sunt Galli.
*Minor*—Omnes Galli sunt aves.
*Ergo*—Omnes Parisii sunt aves.—Q. E. D.

No matter how many members the Courts may admit to our Societies, they will have all the immunities and privileges the Legislature, by its enactments, may guarantee them. The number of members has nothing to do with the privileges of those members. There can be no organization having a legal existence, over which the judiciary shall not have jurisdiction when appealed to by individuals who claim to have been wrongfully excluded from its benefits. They possess and exercise this power over all organizations, religious and secular. Individuals expelled from Christian churches have been re-instated by the Courts;* yet no one claims that membership of a church confers no benefits, or that Christianity holds out no hope of Heaven. This supervisory jurisdiction of the Courts is a very necessary safeguard of our liberties, and however much we may dislike it as physicians, we are bound to uphold and defend it as patriots. But it does not seem to me that our Courts have, as a rule, shown any disposition to relax the conditions of honorable standing in the profession. Not that they may not have rendered wrong decisions, and been the means of forcing upon us unworthy associates, but I believe every such case has arisen from a misapprehension of facts, rather than from an unworthy animus. I should be loth to believe that our judiciary, or even a majority of the members of the legal fraternity, were in favor of supporting quackery, or wished in any way to derogate from the true dignity of our profession. And in support of this position I may cite you to a decision published in your August number, which, whatever may be its justice as to matters of

* This of course applies to the *temporal*, not the *spiritual* affairs of the church.

fact, certainly affirms in clear terms the right and duty of our Society to jealously guard its doors from unworthy intruders. There is too much parallelism between the two professions to permit us to suppose our Judges in any degree leagued with, or in favor of, quackery. And the manner in which they have maintained the dignity of their profession, amid similar discouragements to those we have labored under, merits our respect. Both professions have been made the subject of Legislative interference, and it is to that body we must address our grievances. I am not writing to defend the Courts, but to show that if our field has been made common property it is rather because the Legislature has torn down the fence than because the Courts have opened too wide the gates. Let us transfer then the *onus opprobrii* from the expounders, to the makers of our laws, and your argument will at least be logical in its sequence, whatever may be thought of its validity. Viewed in this light your argument is the same in effect, which has been used against the County Societies ever since the sweeping enactments of the Legislature of 1844. Previous to that year our profession had been hedged about by a high wall of law which effectually prevented access to its ranks without due preparation, and at least the semblance of fair fame. Yet I should be sorry to believe that there is not as much honor and dignity among us now as among our predecessors then. But much misapprehension exists as to the nature of the change made in our legal condition by the laws of that year, While it is true that any person may practice medicine who can get patients to employ him, it is not quite true that ignorant pretenders are placed by the law upon an equal footing with regularly educated physicians, On the contrary the distinction between these two classes is distinctly made and maintained. An irregular practitioner is liable for mal-practice to both criminal and civil prosecution; a regular physician to the latter only. A regularly qualified physician may teach his profession—an irregular cannot. By the terms of the charters of the various colleges, (including I believe the Homœopathic and Eclectic,) certificates of study with a "physician duly qualified to practice his profession" are necessary preliminaries to the reception of a degree. Though as philanthropists, as well as physicians, we ought to desire that no one should be allowed to interfere with the human frame unless duly educated and qualified, we are not to conclude too hastily that all is yet lost. I cannot do better than to quote here the conclusion of a report made to the Kings County Medical Society and published in the transactions of the State Society in 1858. It says: "But what positive advantages, it is asked, remain to those who comply with the requirements of the law? We answer, the advantages of safety and honor, of protection and privilege. The consciousness of rectitude, possibly the opportunity and means of improvement given or received. In short, all that was ever connected with these Societies, except the monopoly of the trade."

Do not imagine that I have even the slightest desire to defend our lawmakers from your just indignation for a course of legislation which seems admirably calculated to exalt quackery, and delusion, at the expense of the legitimate medical profession; far be it from me to undertake so ungrateful a task. But I do maintain that they have not been successful in this end. I maintain that the honor and dignity of our profession are things innate, and inherent, and far above and beyond all legislation. No man shall go beyond me in upholding the true nobility of our calling, or in striving to keep its ranks free from unworthy intruders, but I cannot believe that prohibitory laws constitute the only means of purification, or confer the only dignity and honor. If it be true that our County Societies "protect no interests and teach no medical or other science," then it may be pertinent to ask what has become of the spirit of professional courtesy and pride which ought to exist among their members? If our edifice is yielding to the assaults of quackery, something must have been lacking in our defence, and we may not unprofitably ponder upon the language of the ancient orator:

"*Nos antem viri fortes satisfacere reipublicae videmur, si istius furorem ac tela vitemus,*" (for *reipublicae* read *societies.*) We must have been too much intent upon individual gain, and too little upon those interests of the whole fraternity which ought to be our jealous care.

But I deny the conclusion, and on the contrary maintain that the County Society is yet an institution of both honor and dignity. If not, why this anxiety to get into it? Were it a mere dead body the courts would not spend their time adjudicating in regard to it, nor would any man waste his money trying to force an entrance into it.

You advocate the death and burial of these Societies. Sir, I answer, suicide is the coward's resource, from evils he fears to meet. Let us rather like brave men, resolve to *live*. If the law will not grant us dignity let us take it by deserving it. The dignity of our profession rests upon the individual worth of its members. Let us then strive to raise the standard of that worth by every means in our power. You testify to the efficacy of voluntary association for that purpose, then why abandon any one of the Societies we have already established? To quote a homely proverb, "there are black sheep in every flock." If unworthy men are foisted upon us, let us try to raise them to a level of high moral excellence and worth, resting assured if we fail they will sink to oblivion by the force of their own gravity, and we shall be in no degree lowered by their fall. Good men are none the less good because bad men wear the same form, and it is but a fool's argument against Christianity to assert that sinners sometimes

"Steal the livery of Heaven to serve the devil in."

P.

We are most happy to publish the above communication, and glad to have the jurisdiction of the judiciary so grandly glorified, and to know that the County Societies are institutions of such honor and dignity. We should like to inquire if at the millennium the Courts will not admit sinners to the fellowship of the churches without baptism or confession? It will probably be the "right and duty" of men to be christians; the jurisdiction of the judiciary is established "they possess and exercise this power over all organizations, both religious and secular."

Again we should like to know how it is that we have our incorporated Eclectic and Homœopathic Medical Colleges, if irregular practitioners cannot legally teach their professions? There are many other things we should be willing to know, but as they have no remote bearing upon the value of the County Societies, or the legitimate jurisdiction of the Courts, we will not now stop to make inquiry.

We see very little in the above communication which we cannot heartily endorse, and nothing which tends towards controverting the main and essential propositions advocated in our editorial article.—Ed.

